# Growing (Up) in Times of Multiple Crises – A Call for Mental Health (Research) Action

**DOI:** 10.32872/cpe.12713

**Published:** 2023-09-29

**Authors:** Julia Asbrand, Tanja Michael, Hanna Christiansen, Gerhard Reese

**Affiliations:** 1 Department of Psychology, Friedrich-Schiller-University Jena, Jena, Germany; 2 Department of Psychology, Saarland University, Saarbrucken, Germany; 3 Department of Psychology, University of Marburg, Marburg, Germany; 4 Deutsches Zentrum für Psychische Gesundheit (DZPG), Bochum/Marburg, Germany; 5 Department of Psychology, RPTU Kaiserslautern-Landau, Campus Landau, Landau, Germany

The rock band *Fury in the slaughterhouse* sang that “every generation got its own disease”, however, remaining in that wording, the current generation of children and adolescents in Europe has to cope with several severe “diseases” at the same time: the war of Russia against Ukraine, the social and mental health consequences of the Covid-19-pandemic, the economic downturn, societal polarization, and last but not least, the twin crises of biodiversity loss and climate change.

Each of these crises have visible and measurable consequences, and some crises mutually reinforce each other. The climate crisis, for instance, has already brought irreversible damage to some societies and natural habitats around the world. According to the Intergovernmental Panel on Climate Change (IPCC, 2023), weather phenomena such as droughts, storms and floods will become more likely and more intense. It is estimated that people who are born today will experience heat waves up to seven times more often than people who are in their forties to sixties today (Thiery et al., 2021). Furthermore, climate change is linked to macroeconomic consequences negatively affecting the economic situations of states and individuals and is thus a driving force behind increasing poverty (e.g., Kotz et al., 2021). The climate crisis also accelerates biodiversity loss. Further, both environmental degradation as well as climate change undermine peace and increase the likelihood for conflict between groups, representing additional stressors for development both on an individual and a societal level (e.g., Palmer, 2022). Unsurprisingly, a recent meta-analysis shows that climate events are negatively correlated with mental health (Cuijpers et al., 2023), and a recent review demonstrates that the risk for mental health problems among young people is particularly high (Ma, Moore, & Cleary, 2022).

The Covid-19 pandemic decreased mental health in the general population, and younger age groups in particular (Santomauro et al., 2021). Furthermore, several studies show that the worsened mental health of young people remained up to two years after the onset of the pandemic (Hansen et al., 2023). A recent study among German adolescents also shows that both pandemic-related and climate-related distress are linked to more depression and anxiety symptoms and to reduced health-related quality of life. Distress related to the Russia-Ukraine war was associated with greater anxiety. Critically, these associations remained significant when controlling for important covariates (e.g., gender, distress caused by personal problems), showing that the crisis measures have incremental predictive value (Lass-Hennemann et al., 2023). However, self-efficacy and, though to a lesser extent, expressive flexibility were associated with better mental health. There are no studies yet examining how the war influences mental health of children and adolescents living in an area directly exposed to the war.

Despite the increased need to address mental health problems among young people (e.g. Deng et al., 2023), sufficient in-patient and outpatient mental health care systems are yet to be implemented. Actually, waiting times have doubled during the pandemic and low-threshold effective interventions are lacking (e.g. Overhage et al., 2023).

In sum, the current evidence suggests that global crises impact the mental health and healthy upbringing of young people. Therefore, policies should include interventions that help children and adolescents in particular to cope with the stress caused by the crises.

## The Systemic Structure Underneath: What Can We (Not) Do?

However, it is not enough to only develop strategies to help individuals to cope better with stress or to increase mental health care capacities. Large scale crises, as the ones depicted above, have in common that they are usually a result of collective (in-)action and as such, these crises cannot be remedied by individual action alone: As an individual, I can neither address the pandemic, solve the climate crisis nor end a war all by myself. In other words: An individual can hardly experience self-efficacy when faced with these challenges.

These challenges can only be addressed by collective efforts, and these efforts must be implemented on various societal levels. For example, the multilevel model of societal change introduced by Geels and Schot (2007) represents a framework that helps to understand how and which levels of society need to be addressed to achieve societal change. In short, the model suggests that certain pressures such as climate change or resource scarcity open windows of opportunity within a political regime. Networks of innovators and groups with joint ideas can then use such a window to engender change within society. For example, the Fridays-for-Future movement did so, and changed the way climate change is treated in politics and society. Recent psychological models suggest the pathways through which collective and participatory efficacy beliefs can foster such collective actions (Hamann et al., 2023).

Building on these systemic considerations, clinical psychology and psychotherapy, both at the level of care and research, urgently need to move away from an exclusively individual approach to a consideration of the individual in the system, its structures, and their relevant life-environments such as schools or the work-place. How could this look like?

## A Call for Health(y) Action

First of all, broader prevention structures focusing on systemic levels of mental health as well as self-efficacy (Lass-Hennemann et al., 2023) and adaptive coping are necessary (Mah et al., 2020). These need to be implemented and institutionalized within structural levels, and have to provide an outreach service – independent of youth’s knowledge about health care structures or individual resources. Second, these prevention structures must also be flanked by measures that mitigate major risk factors for mental health and a negative trajectory when growing up. Specifically, these are all measures targeting financial and social injustices and inequity, i.e. installing appropriate climate protection measures, providing access to education and living above the poverty line is part of health care. Third, our research must do justice to the complex interplay between the individual and society, also in the field of mental health. This means that research approaches must be promoted in clinical psychology and psychotherapy that situate the individual in society and clarify the effects of society on the individual, e.g. which systemic structures limit treatment success? How do societal crises affect mental health?

We believe that the current age of multiple crises holds several challenges for mental health care and research, foremost the fact that we are affected by these crises as health care professionals and researchers. To adequately address these challenges, we need to expand (preventive) healthcare, intensify our research efforts and at the societal level to help young people grow up healthy.
